# Healthier Diet and Diet-Related Behaviors Are Associated with Increased Physical Activity and Reduced Sedentary Behavior Among Adolescents in Greece

**DOI:** 10.3390/nu17030381

**Published:** 2025-01-21

**Authors:** Eleftheria Chaireti, Philippos Orfanos, Anastasios Fotiou, Eleftheria Kanavou, Myrto Stavrou, Clive Richardson, Anna Kokkevi, Vassiliki Benetou

**Affiliations:** 1 Department of Hygiene, Epidemiology and Medical Statistics, School of Medicine, National and Kapodistrian University of Athens, 115-27 Athens, Greece; elefthch@med.uoa.gr (E.C.); phorfanos@med.uoa.gr (P.O.); 2 University Mental Health, Neurosciences, & Precision Medicine Research Institute “Costas Stefanis” (UMHRI), 115-27 Athens, Greece; afotiou@med.uoa.gr (A.F.); eleftheria.kanavou@gmail.com (E.K.); myrtostavrou@gmail.com (M.S.); akokkevi@med.uoa.gr (A.K.); 3 Department of Economic and Regional Development, Panteion University of Social and Political Sciences, 176-71 Athens, Greece; crichard@panteion.gr

**Keywords:** adolescents, diet, physical activity, sedentary behavior, screen time, recommendations

## Abstract

Background/Objectives: Healthy diet, regular physical activity (PA), and minimizing sedentary behavior (SB) are crucial in promoting adolescents’ health and well-being. We assessed adherence to PA and SB recommendations among a representative sample of adolescents and explored their relationship with diet and diet-related behaviors. Methods: Data from the Greek arm of the 2017/2018 international Health Behavior in School-Aged Children (HBSC) cross-sectional study were used, including a probability sample of 3357 students (47.6% boys) who were 11, 13, and 15 years old. PA, SB, consumption of food groups/beverages, and diet-related behaviors were self-reported. Multiple logistic regression was used to identify potential associations. Results: Most students failed to meet PA (83.9%) (i.e., ≥1 h of moderate-to-vigorous physical activity/day) and SB (90.2%) (i.e., ≤2 h of screen time/day) recommendations. Daily consumption of fruit and vegetables compared to consumption at ≤1 day/week was associated with increased adherence to PA recommendations (adjusted Odds Ratio (aOR) = 2.26, 95% Confidence Intervals (CI): 1.62–3.17 for fruit; aOR = 1.35, 95%CI: 1.00–1.82 for vegetables). Eating sweets ≤1 day/week vs. every day was associated with higher adherence to SB recommendations (aOR = 2.41, 95%CI: 1.43–4.04). Poor diet quality was related to lower adherence to PA and SB recommendations. Daily breakfast consumption vs. never and abstaining from eating at fast-food restaurants were associated with better adherence to PA and SB recommendations. Rarely eating in front of screens was associated with substantially higher odds of adhering to SB recommendations (aOR = 5.79, 95%CI: 3.67–9.14). Conclusions: Healthier diet/diet-related behaviors were associated with increased PA and reduced SB in this sample of adolescents.

## 1. Introduction

Physical activity, sedentary behavior, and diet are considered to be among the most significant modifiable health determinants for children and adolescents [1,2,3]. The indisputable impact of physical activity (PA) on children’s and adolescents’ health has led the World Health Organization (WHO) to publish evidence-based public health recommendations on the intensity, frequency, and duration of physical activity for young people in order to achieve significant health benefits and mitigate health risks. Thus, current global recommendations state that children and adolescents aged 5–17 years old should accumulate at least an average of 60 min of moderate to vigorous intensity physical activity per day [1]. Furthermore, given the recognition of the negative effects of sedentary behavior (SB) and the growing body of data revealing the “sedentarism pandemic” among young people [2], the WHO guidelines included SB recommendations separately from those on physical activity for the first time, which recommend limiting the amount of sedentary time, in particular, recreational screen time, without setting a specific time limit [1]. On the other hand, several national guidelines worldwide (e.g., Canadian, American, Australian) have recommended limiting recreational screen time to no more than 2 h per day for children and adolescents 5–17 years of age [4,5,6]. The American Academy of Pediatrics further acknowledged that an individualized approach, rather than a one-size-fits-all one, and the quality of interaction with digital media are important in setting a screen time limit [7]. Most European Union (EU) member States, as well as countries of the WHO European region, follow the WHO guidelines for physical activity and sedentary behavior, whereas current Greek national guidelines for children and adolescents have further set the limit of two hours in front of screens for this age group [8,9,10]. Also of note is that sedentary behavior is defined as any waking activity characterized by low energy expenditure; more specifically at ≤1.5 metabolic equivalents (METs) while in a sitting, reclining, or lying posture (e.g., watching TV, gaming/working on PC, reading, etc.) [11].

Despite the recommendations issued by the WHO and international and national scientific societies, data on physical activity and sedentary behavior levels among young people worldwide are disturbing. A pooled analysis of 298 school-based surveys with 1.6 million students aged 11–17 years found that, in 2016, more than 80% of adolescents worldwide did not meet the recommendations for daily physical activity [12]. In Europe, and in a pooled analysis with harmonized, accelerometry-measured physical activity data from studies conducted in 18 European countries between 1997 and 2014, at least 70% of the adolescents were categorized as insufficiently physically active, with a significant north–south gradient for total PA and moderate-to-vigorous physical activity (MVPA); adolescents living in Southern Europe practice MVPA on average 5 min less per day than their peers in Northern Europe, irrespective of age and the body mass index (BMI) [13]. With respect to sedentary behavior, global data show that adolescents spend almost 60% of their waking hours seated [14], whereas about two-thirds of the youth population consistently exceed 2 h of daily screen time [15]. A recent systematic review and meta-analysis indicated an increase in sedentary time as children and adolescents age; an increase of approximately 28 min over 1 year and 141 min over 4 years of follow-up was reported, with no differentiation in the pattern of change according to age or sex [16].

Diet and movement behaviors are intrinsically related through their involvement in energy balance and body weight (often called energy-balance-related behaviors) and their complex interactions, which significantly impact health [17]. Dietary habits, as well as movement behaviors, are formed early in life, and following a healthy diet is critically important for promoting health and well-being throughout the course of life. Currently, dietary recommendations for apparently healthy adults and children aged two years and above advocate the adoption of a sustainable diet based mostly on a variety of plant-based foods (i.e., vegetables, fruit, pulses, unrefined cereals) and less on animal-based foods, with a preference for unsaturated rather than saturated oils and fats and small amounts of refined grains, highly processed foods, and added sugars [3]. Diet-related behaviors during childhood and adolescence, such as regular breakfast consumption, eating with the family, and avoiding eating in front of screens, have also been associated with better health and normal body weight [18].

Previous studies have demonstrated an association between physical activity, sedentary behavior, and dietary habits among children and adolescents [19,20,21,22,23,24,25]. Chacón-Cuberos et al. reported that engagement in PA for more than three hours per week in a sample of Spanish adolescents was related to higher levels of adherence to the Mediterranean diet (MD) [19]. More physically active Iranian adolescents were more likely to consume fruit, vegetables, and dairy products, compared to their less physically active peers [20]. Similarly, data from the NYPASS study of US high school students showed that meeting PA recommendations was associated with a higher intake of fruit and vegetables, whereas exceeding limits of daily screen time was related to a lower intake of fruits and vegetables and a higher intake of sweets and sugar-sweetened beverages [21]. Other studies among children and adolescents have also found an association between higher levels of physical activity, a healthier diet, and/or reduced sedentary behavior [22,23,24,25].

Furthermore, it has been suggested that diet, PA, and SB tend to cluster in both healthy and unhealthy ways. Cluster patterns seem to differ by age, gender, and socioeconomic status; older age and low socioeconomic status have been consistently represented in the unhealthier clusters, whereas findings regarding gender are not so consistent [24,26]. The presence of mixed behavior clusters with the coexistence of healthy and unhealthy behaviors, e.g., high levels of PA, increased screen time, and high consumption of ultra-processed foods (i.e., snacks, sugar-sweetened beverages), has been frequently observed among children and adolescents [26].

Concerning sedentary behavior, a systematic review of observational studies indicated a clear association of sedentary behavior, with higher consumption of energy-dense snacks, drinks, and fast foods, in conjunction with lower consumption of fruit and vegetables, among children and adolescents and also among adults [27]. Moreover, the international ISCOLE study among children 9–11 years of age from 12 countries identified a link between a healthier dietary pattern and the achievement of movement behavior recommendations (screen time, physical activity, and sleep duration); among the three behaviors, fulfilling the screen time recommendations was the one most strongly linked to healthier dietary patterns [28].

Greece faces significant challenges with high rates of overweightness and obesity among its adolescents, which rank among the highest in Europe [29]. This issue is further complicated by prevalent sedentary behaviors, such as excessive screen time, which are associated with adverse metabolic and psychological outcomes [30,31]. Furthermore, studies indicate that adherence to healthy dietary patterns (e.g., Mediterranean diet) and national dietary recommendations is low among adolescents in Greece [32]. Data from a representative sample of 177,091 students, aged 8–17 years old, living in Greece in 2015, showed that healthier dietary habits and acceptable screen time use were strongly associated with adequate PA levels [33]. Other studies, with participants from Greece, have also investigated physical activity and sedentary behavior in relation to diet, but they have focused on their relationship with obesity and with sociodemographic factors, mostly among preschoolers [34] or among children in need [35].

Overall, relatively few studies have examined the relationships between diet, physical activity, and sedentary time concurrently among children and adolescents, both in Greece and globally. Understanding these associations is essential for designing effective interventions and programs that promote the adoption of a healthy, active lifestyle during adolescence—a critical period when habits often persist into adulthood and influence long-term health outcomes [36].

Based on the above, the aim of the present study was to explore the association between dietary habits and behaviors and adherence to recommendations on physical activity and sedentary behavior in a large sample of 11, 13, and 15-year-old students living in Greece. We hypothesized that adolescents with healthier dietary habits and behaviors would be more likely to meet international recommendations on regular physical activity and reduced sedentary time.

## 2. Materials and Methods

### 2.1. Study Design

Data on PA, SB, and diet-related habits and behaviors were derived from the Greek arm of the 2017/2018 international Health Behavior in School-Aged Children (HBSC) study, conducted in Greece by the University Mental Health, Neurosciences, and Precision Medicine Research Institute (UMHRI) situated in Athens, Greece. The HBSC study is a unique, multinational, cross-sectional survey conducted quadrennially since 1983/1984 across Europe and North America. It is in collaboration with the WHO Regional Office for Europe, with the aim of gaining insight into adolescent health and well-being and informing policy and practice to improve young people’s lives. Each survey uses a standard methodology detailed in the HBSC common research protocol in order to achieve high quality and comparability of the data as well as representative study samples [37].

### 2.2. Study Population

Students aged 11, 13, and 15 years old attending school at the time of the administration of the survey were eligible to participate in the study. No exclusion criteria were in place. Thus, students from the 6th, 8th, and 10th grades were selected using a one-stage stratified random cluster sampling design, which is in line with the 2017/2018 HBSC study protocol, in order to achieve a nationally representative sample based on the country’s administrative regions and the school type (comprehensive/vocational, public/private). The sampling unit was the school class, and all registered students in the sampled classes were eligible to participate after passive parental consent. Out of the selected schools, 238 responded (a response rate of 93%), and 87% of the attending students agreed to participate in the survey. The anonymous, self-completed questionnaire was administered in the classroom by trained research assistants. Ethical approval was obtained from the Greek Ministry of Education (approval codes: 25535-D1 and 41305-D2). From the initial sample of 3863 students, who were participants in the 2017/2018 survey, 506 were excluded because of a high proportion of missing values in the variables used in this analysis (except for the BMI). Thus, the final study sample consisted of 3357 students, 1597 (47.6%) boys and 1760 (52.4%) girls, aged 11, 13, and 15 years old.

### 2.3. Survey Instruments and Measurements

A Greek version of the HBSC 2017/2018 standardized questionnaire was used. The validity and reliability of key items in this instrument have been examined in the past with satisfactory results [38,39,40,41].

### 2.4. Information on Physical Activity 

Physical activity was assessed using the moderate-to-vigorous physical activity (MVPA) item. Participants were asked to report how many days during the past week they were physically active at a moderate-to-vigorous intensity level (providing examples, such as running, biking, dancing, swimming, soccer, basketball, school activities, etc.) for a total of at least 60 min. The item has been shown to have a reasonable validity and significant correlation with accelerometer data [42,43], as well as acceptable test–retest reliability when used as a dichotomous variable [44].

### 2.5. Information on Sedentary Behavior

The measure of sedentary behavior, which was available in this questionnaire, used three items to capture typical, screen-based, and sedentary activities during free time: (a) Watching television (including videos/YouTube, DVDs, etc.); (b) Gaming on screens (e.g., on a computer, games console, tablet (like iPad), smartphone, or other electronic device), not including games involving movement or exercise; and (c) Using electronic devices, such as computers, tablets (like iPad), and smartphones, for other purposes (e.g., homework, emailing, chatting, surfing on the internet). Answers were given on a 9-point scale ranging from “none at all”, “about half an hour a day”, “about an hour a day”, and hourly intervals thereafter up to ≥7 h daily. The items were answered separately for weekdays and weekends, and an average daily sedentary time was calculated as a weighted mean. Validation studies have supported the acceptable test–retest reliability of these items and convergent validity with a weekly self-report TV diary [45,46].

### 2.6. Food Consumption-Diet Quality Score

Students’ dietary habits were assessed using the HBSC food frequency questionnaire (FFQ), which includes four food groups and beverages—fruit, vegetables/salads, sweets, and sugar-sweetened beverages (SSBs)—all considered key items for adolescent diet. The 7-point answer scale was merged into four categories for each food group for the present analysis; “once a week or less”, “2–4 days a week”, “5–6 days a week”, “at least every day”. Following the recommendations of the Greek National Food-based Dietary Guidelines for children and adolescents, a diet quality score was also generated [9,47]. For each of the four food groups of the FFQ, a 5-point scale (values 0–4) was used based on the consumption frequency (in days/week) and the assumed diet quality of each food group. For food items (i.e., vegetables/salads and fruit) that are recommended as part of a healthy everyday diet, higher values represented more frequent consumption, whereas, for food items that are not recommended as part of a healthy everyday diet (i.e., sweets and SSBs), higher values represented less frequent consumption. The score could take values from 0 to 16, with higher values depicting better diet quality. A categorical variable was generated based on the dietary score’s distribution tertiles as follows: good diet quality score (points 13–16), moderate (points 10–12), and poor diet quality score (points 0–9).

### 2.7. Diet-Related Behaviors

Six diet-related behaviors were evaluated through the questionnaire: (a) Breakfast consumption on weekdays; (b) Family meals; (c) Eating in fast-food restaurants; (d) Eating snacks while watching TV; (e) Eating snacks while sitting in front of screens; (f) Eating meals while watching TV. With respect to breakfast consumption, students were asked to report how many schooldays during the week they had breakfast (defined as having more than one glass of milk or fruit juice) and their answers were classified into three categories: “never”, “1–4 schooldays/week” and “5 schooldays/week”. For recording frequency of eating with the family, participants were grouped based on their responses, into two categories “once a week or less” and “most days”, while for frequency of eating in fast-food restaurants, students were grouped into four categories: “at least once a week”, “1–3 days a month”, “less than once a month” and “never”. Concerning the last three diet-related behaviors (d–f) associated with screen-linked activities, a combined score was also developed, with higher scores depicting a higher frequency of eating in front of screens, which was further categorized into three groups: “most days”, “less often”, and “rarely” and used as a categorical variable.

### 2.8. Information on Other Covariates

Participants’ sociodemographic data, including age, gender, country of birth (personal and parental), and school region were also collected. Anthropometric data, and more specifically, height (in meters) and body weight (in kgs), were collected from students’ self-reports. The body mass index (BMI) was calculated as the ratio of weight (in Kg) divided by the square of height in meters (kg/m^2^), and age- and gender-specific z-scores were calculated. Students were classified into categories (underweight, normal weight, overweight, and obese) based on their BMI-for-age according to the 2007 WHO growth charts [48]. Family socioeconomic status was assessed using the family affluence scale (FAS-III), which evaluates absolute affluence through six common material assets or activities (number of computers and cars, separate student’s bedroom, number of home bathrooms, ownership of dishwasher, frequency of family holidays abroad). The total score was used to classify adolescents into three relative affluence groups (lowest 20%, middle 60%, and highest 20%). Adherence to age-specific sleep duration recommendations, according to the American Academy of Sleep Medicine guidelines [49], and students’ life satisfaction level (scale of 0–10, with “10” corresponding to the best possible life students could have), as well as their weight reduction behavior, were also recorded.

### 2.9. Statistical Analyses

Descriptive statistics were calculated using absolute and relative frequencies for categorical variables and means and standard deviations for continuous variables. Differences between the groups were determined via a two-sample Wilcoxon rank-sum test for the continuous variables with non-normal distribution and via Pearson’s chi-square test (χ^2^) for categorical variables. The phi (φ) coefficient was applied for 2 × 2 contingency tables, providing a correlation-like measure ranging from −1 (perfect negative association) to +1 (perfect positive association). For contingency tables larger than 2 × 2, Cramér’s V, derived from the chi-square statistic, was used to quantify the strength of association, ranging from 0 (no association) to 1 (perfect association). While the phi coefficient provides information on both the strength and direction of the association, Cramér’s V quantifies only the strength of the association without indicating direction [50].

Physical activity level and sedentary behavior were defined as dependent categorical variables for this analysis. A dichotomous variable was generated for physical activity in order to identify students meeting (i.e., ≥1 h MVPA daily) and not meeting (i.e., <1 h of MVPA daily) the WHO recommendations for PA. For sedentary behavior, a dichotomous variable distinguished students at the cut-off of 2 h/daily spending on screen-based sedentary activities, those meeting the recommendations (i.e., ≤2 h daily) and those not meeting the recommendations (i.e., >2 h daily). Regarding the BMI, the overweight and obese categories were unified into one group (overweight group), and normal weight and underweight into another (non-overweight group), with a third category consisting of participants with missing values for the BMI (due to a high number of missing values for this variable).

Binary logistic regression models were used to estimate odds ratios (OR) and 95% confidence intervals (CI) for associations between adherence to PA and SB recommendations and dietary variables. A multivariable model including the four individual food-groups and beverages and the available variables of diet-related behaviors, adjusted for all sociodemographic and baseline characteristics (gender, age groups, students’ and parents’ place of birth, BMI z-score groups, family affluence score groups, life satisfaction level, dieting, and sleeping habits) was first calculated for the total sample, separately for PA (Model 1) and SB (Model 2). Multivariable models were also stratified by gender. Finally, multivariable analyses were repeated after replacing individual food groups and beverages with diet-quality groups (Model 3).

For better interpretation and for the identification of dietary habits and behaviors that are associated with increased odds of meeting the PA and SB guidelines, we set as baseline categories those that indicated non-compliance with the recommended PA and SB recommendations; thus, an odds ratio (OR) greater than 1 indicated a factor positively associated with meeting the PA and SB recommendations. Statistical significance was set at *p*-value < 0.05, and analyses were conducted using STATA v13.1 (STATA Corporation, College Station, TX, USA), accounting for the survey design.

## 3. Results

Table 1 shows participants’ sociodemographic and other characteristics recorded at baseline. Students were evenly distributed across the 11-, 13- and 15-year-old age groups. More than half of them (52.9%) resided in the prefectures where the capital of Greece, Athens, and the second largest city, Thessaloniki, are situated. Also, the vast majority were born in Greece (96.7%), whereas almost 25% of them had at least one of their parents born outside Greece. Nearly 84% of the adolescents failed to meet the WHO recommendations for daily PA levels. On average, participants engaged in moderate-to-vigorous intensity physical activity (MVPA) for at least 60 min/day, for 4 days per week. Concerning sedentary behavior (SB), 90.2% of adolescents exceeded the total daily sedentary time limit of ≤2 h, averaging 6.5 h (390 min) daily in the total sample (Table 1).

In Table 2, sociodemographic and baseline characteristics by fulfillment of physical activity (no/yes) and sedentary behavior recommendations (no/yes) are shown.

Boys met the daily recommended 1 h MVPA more than girls (20.1% vs. 12.4%) and the youngest students more than the oldest (20.9% among 11 year olds vs. 12.6% among 15 year olds). Non-overweight adolescents were more likely to meet PA recommendations compared to their overweight/obese peers (17.1% vs. 12.7%). Students in the high family affluence scale category were more likely to adhere to PA recommendations compared to students in the middle and low categories. Concerning sedentary behavior, a higher percentage of boys exceeded the recommended threshold compared to girls (92.1% vs. 88.5%) and the oldest participants compared to the youngest (94.2% of the 15 year olds vs. 81.3% of the 11 year olds). Students with higher life satisfaction scores adhered more to physical activity and sedentary behavior guidelines. Students adhering to age-specific sleep duration recommendations were more likely to meet SB guidelines compared to those not meeting the sleep recommendations (13.5% vs. 6.7%). No significant differences in the BMI were observed between adolescents meeting and not meeting sedentary time guidelines.

Table 3 presents the frequency of consumption of specific food items/beverages and other diet-related behaviors in the total sample, as well as by adherence to physical activity and sedentary time, according to international recommendations.

Students who met the WHO guidelines for MVPA exhibited higher daily fruit and vegetable consumption compared to their less active counterparts (48.0% vs. 27.4% for fruit; 44.8% vs. 31.9% for vegetables), and lower daily sweets consumption (14.3% vs. 15.2%). Similarly, those meeting sedentary time recommendations reported higher daily fruit and vegetable consumption than their non-compliant peers (45.4% vs. 29.1% for fruit; 41.5% vs. 33.2% for vegetables). Conversely, students with prolonged sedentary behavior (>2 h daily) reported more frequent consumption of sweets and SSBs than their less inactive counterparts (16.1% vs. 5.4% for at least daily consumption of sweets, 5.6% vs. 2.1% for at least daily consumption of beverages, respectively). Approximately one-third of adolescents adhering to PA (31.3%) and SB (34.5%) guidelines were grouped into the good diet quality category, in contrast to approximately 16% of the least adherent students (15.7% for PA and 16.5% for SB).

Concerning diet-related behaviors, almost half of the students reported having breakfast every weekday (47.8%), yet 28.0% never consumed breakfast on weekdays. Students not meeting the PA recommendations were more likely to never eat breakfast in weekdays, compared to their active counterparts (28.7% vs. 24.4%). Similarly, students not meeting the sedentary time limit recommendations were more likely to skip breakfast compared to those who adhered to them (29.1% vs. 17.9%). Eating with the family was almost a daily habit for 79.9% of the participants, more prevalent among those meeting SB recommendations compared to those who did not (86.1% vs. 79.2%). Additionally, fast-food consumption at least once per week was significantly more common among students not adhering to international guidelines for PA and SB, compared to those adhering (24.4% vs. 20.9% for PA and 25.2% vs. 11.2% for SB). Finally, eating in front of screens most days of the week was significantly more common among adolescents with a sedentary lifestyle compared to those with less than 2 h sedentary time daily (35.4% vs. 11.2%) (Table 3). The strength of the associations shown in Table 2 and Table 3 was generally weak based on Phi/Cramer’s V measure.

Associations between dietary habits, diet-related behaviors, and adherence to international guidelines for PA and SB derived from the multiple logistic regression are presented in Table 4 and Table 5, respectively.

### 3.1. Associations Between Dietary Habits and Behaviors and Adherence to PA Recommendations

Girls exhibited nearly 50% lower odds of meeting international PA guidelines compared to boys (aOR = 0.49, 95%CI: 0.40–0.62). Among girls, age was inversely associated with PA, with 15-year-old females having lower odds of achieving recommended PA durations compared to 11 year olds (aOR = 0.52, 95%CI: 0.35- 0.79).

Frequency of fruit consumption was significantly associated with adherence to physical activity recommendations for both genders; more specifically, daily fruit consumption, compared to consumption once per week or less, was associated with 68% higher odds of meeting the PA recommendations among girls (aOR = 1.68, 95%CI: 1.02–2.73), and almost 3 times higher odds among boys (aOR = 2.95, 95%CI: 1.92–4.53). Also, daily vegetable intake, compared to once per week or less, was associated with 35% higher odds of meeting the recommended duration of PA in the total sample (aOR = 1.35, 95%CI: 1.00–1.82), but no significant associations were found when stratified by gender. Consumption of sweets and SSBs were not significantly associated with meeting the physical activity recommendations.

Regarding diet-related behaviors, boys consuming breakfast every weekday (aOR = 1.55, 95%CI: 1.12–2.14) and those abstaining from fast food restaurants (aOR = 2.12, 95%CI: 1.15–3.88) had 1.5-fold and almost 2-fold increased odds of adhering to PA guidelines compared to those never eating breakfast and those eating in fast food restaurants on a weekly basis, respectively (Table 4). Overweight students exhibited almost 50% and 40% lower odds of achieving the recommended PA recommendations compared to their non-overweight counterparts, for girls and boys, respectively. Finally, higher life satisfaction levels and dieting to lose weight were also associated with increased odds of meeting PA recommendations among female students.

### 3.2. Associations Between Dietary Habits and Behaviors and Adherence to Sedentary Behavior Recommendations

Girls demonstrated almost 50% higher odds of meeting international guidelines for SB, compared to boys (aOR = 1.43, 95%CI: 1.09–1.89) (Table 5). Additionally, older students, regardless of gender, exhibited reduced odds of adhering to SB guidelines compared to younger peers (aOR = 0.33, 95%CI: 0.20–0.53 for boys; aOR = 0.29, 95%CI: 0.18–0.47 for girls aged 13 compared to aged 11).

In terms of dietary habits, girls consuming sweets once per week or less had significantly higher odds of engaging in less than 2 h of sedentary activities per day, compared to those who consumed sweets on a daily basis (aOR = 2.46, 95%CI: 1.25–4.85). None of the other food groups were associated with meeting sedentary time limit recommendations. Consuming breakfast every weekday was associated with almost a 2-fold increase in the odds of complying with SB recommendations compared to always skipping breakfast among both girls (aOR = 1.86, 95%CI: 1.20–2.87) and boys (aOR = 1.70, 95%CI: 1.02–2.85). Eating rarely in front of screens compared to almost every day was associated with a 6-fold increase in odds of complying with SB recommendations among both genders (aOR: 5.92, 95%CI: 3.16–11.11 for girls; aOR = 6.01, 95%CI: 3.08–11.72 for boys). Girls who never ate in fast-food restaurants had nearly 3 times higher odds of adhering to SB recommendations compared to those consuming fast-food every week (aOR = 2.74, 95%CI: 1.22–6.15), with no significant associations among boys (Table 5). Adherence to age-specific sleep duration recommendations was associated with nearly a twofold increase in the odds of engaging in SB recommendations, while each unit increase in the life satisfaction scale was associated with a 22% increase in the odds of adhering to SB guidelines among boys. No significant associations were noted for the BMI and weight reduction behaviors.

### 3.3. Associations Between Diet Quality and Adherence to PA and SB Recommendations

Poor diet quality was associated with lower adherence to PA and SB recommendations (Table 6). Thus, students with poor diet quality had more than 60% lower odds of being compliant with PA recommendations in both genders (aOR = 0.35, 95%CI: 0.24–0.51 for boys, aOR = 0.38, 95%CI: 0.24–0.60 for girls), and almost 30% lower odds of adhering to SB guidelines. However, regarding SB, statistical significance was only observed in the total study sample and among girls, not among boys (aOR = 0.67, 95%CI: 0.47–0.94 for the total sample, aOR = 0.63, 95%CI: 0.41–0.97 for girls).

## 4. Discussion

The present study examined the association between individual food groups and beverages, diet quality, and diet-related behaviors with adherence to PA and SB recommendations in a large sample of adolescents aged 11-, 13-, and 15- years old. Adolescents who consumed fruit or vegetables every day compared to those consuming them less often, were more likely to meet PA recommendations. On the other hand, girls who consumed sweets only once per week, or even less, were more likely to meet SB recommendations to limit screen-time. Higher diet quality, characterized by frequent consumption of fruit and vegetables and infrequent consumption of sweets and sugar-sweetened beverages, was associated with higher adherence to recommended levels of both daily PA and SB. Daily breakfast consumption and fewer visits to fast-food restaurants were associated with higher adherence to PA and SB guidelines, whereas frequent eating in front of screens was strongly inversely associated with adherence to SB recommendations.

Adherence to PA and SB international recommendations was very low in this sample of adolescents. In our study, only 16% of students met the WHO guidelines for PA, and over 90% failed to comply with sedentary behavior recommendations. These results are in agreement with the globally recorded trends indicating widespread insufficient physical activity among adolescents [12], albeit with variations across countries and measurement methodologies [51,52]. Consistent also with our results, authors using data from the Sport and Physical Activity EU Special Eurobarometers, collected between 2002 and 2017, concluded that SB, defined as exceeding the limit of 4 h 30 min of total daily sitting time, was extremely high (76.8% in 2017) with no differences between girls and boys [53]. Based on the international report from the 2017/2018 HBSC study, presenting data from over 220,000 young people in 45 countries and regions in Europe and Canada, fewer than one in five adolescents (19%) met the current recommendation of 60 min of MVPA every day; at all ages and in almost all countries/regions, those more likely to be physically active were boys compared to girls, younger adolescents compared to older, and higher-affluence families compared to lower [54]. Among the factors that may have contributed to the observed low physical activity and high sedentary behavior levels during adolescence are increased screen usage, time constraints, low parental activity levels, and limited access to sports facilities [55].

Female gender, older age, and overweight/obese status were significantly associated with lower odds of meeting the international PA guidelines in our study. Gender differences in PA levels have been consistently observed in the literature, with boys typically exhibiting higher activity levels than girls [13,52,53]. Perceived barriers to physical activity among girls are, among others, the lack of energy, body image, and appearance issues, as well as physical condition [56]. Age-related declines in PA are also well-documented, showing an annual leveling-off of approximately 5%, with a more pronounced decrease observed among girls [54,57,58], possibly due to biological maturity rather than chronological age [59]. The narrow age range (11–15 years) of our study’s participants, coupled with delayed maturity milestones among boys, may account for the absence of a statistically significant age-gradient difference in PA among boys in contrast to girls. Moreover, overweight adolescents consistently demonstrate lower activity levels than their normal-weight peers in the literature, though the direction of causality remains uncertain [60]. Regarding SB, younger age and female gender were associated with higher adherence to sedentary time limit recommendations in this study. The existing literature consistently demonstrates an annual escalation of SB by age among adolescents, estimated at approximately 20 min/day according to previous studies [16]. However, gender differences in SB remain less clear [53,61].

Our findings also revealed a significant association between meeting recommended PA and SB levels and healthier dietary patterns, characterized by frequent consumption of fruit and vegetables/salads and infrequent consumption of sweets and sugar-sweetened beverages (SSBs), assessed by a dietary quality score. Consistent with these results, earlier research has demonstrated a link between PA, SB, and healthier eating habits, particularly adherence to the Mediterranean diet (MD) [62,63,64]. Positive associations of frequent PA and restricted sedentary activity with optimal adherence to an MD dietary pattern have been reported in young populations [64], while data from Greek adolescents have previously shown an inverse relation of adherence to MD with physical inactivity and prolonged engagement in sedentary activities [65]. Proposed mechanisms for these associations include personality traits such as self-regulation, self-efficacy, and intrinsically oriented goals [64], along with the increased need for essential nutrients driven by energy expenditure during PA [20]. Higher exposure to food advertising and snacking during screen time may partly explain the relations between unhealthy food choices and SB [66].

Although an association between adherence to physical activity recommendations and overall dietary quality was observed, the associations were less clear when distinct food groups were examined. Higher PA was linked to increased fruit and vegetable intake in the total sample of our study. Similar associations have also been reported among adolescents from 10 European cities in the context of the HELENA study [67] as well as among German, Spanish, and US adolescents [21,63,68]. No association was identified between adherence to PA recommendations and intake of sweets or sugar-sweetened beverages in the present sample, which is in accordance with other studies [69]. Findings regarding the association between SSBs and physical activity levels in the literature are inconsistent. High intake of soft drinks was associated with lower levels of physical exercise but not with adherence to recommended physical activity levels in a sample of 9842 children and adolescents 6 to 17 year olds, participants in the German Health Interview and Examination Survey for Children and Adolescents (KiGGS Wave 2; 2014–2017) [68]. On the other hand, high consumption of SSBs has also been associated with higher physical activity levels, possibly reflecting a conscious or unconscious attempt to compensate for unhealthy behaviors in one area by adopting healthy behaviors in another, as suggested by some authors [21,26]. Discrepancies in the association may also stem from differences in the types of SSBs examined; for example, Ranjit et al. suggested a decrease in vigorous physical activity levels (VPA) with the level of soda intake and an increase of VPA with the level of flavored and sports beverages intake [70].

Regarding sedentary behavior, higher sweet consumption was significantly associated with increased odds of a sedentary profile among girls in this study. However, no association was evident between fruit, vegetable, or SSB intake and SB. Associations between screen time and dietary habits have been reported by previous studies in Europe and elsewhere. A systematic review reported that higher levels of TV viewing were linked to lower fruit and vegetable consumption and higher SSB intake [71]. Moreover, cross-sectional data among US high school students found an association between daily SSB consumption and prolonged daily screen time [72]. The possible differences in the prevalence of SB type between girls and boys [61] and the higher levels of sedentarism among female participants may have a stronger impact on their dietary choices, explaining the gender differences in the present results. Observed study discrepancies may arise from methodological differences, including definitions of SB (e.g., total screen time versus recreational screen use only) and PA (e.g., total PA, MVPA, or organized sports). In this study sedentary behavior was captured by typical screen-based activities during free time. Additionally, measurement methods (e.g., self-reports vs. accelerometry data) and classification cut-offs for PA/SB recommendations further complicate comparisons. We must note that there is no consensus on absolute screen time limits. The WHO, in its 2020 recommendations, stated that there is not enough evidence to determine a dose–response relationship between sedentary time (including recreational screen time) and health outcomes in children and adolescents and that a precise cut-off for recreational screen time could not be safely determined [31]. Nevertheless, the use of specific threshold values for recreational screen time is useful both for parents and health care providers and has relatively low potential risks. Finally, cultural factors influencing movement and dietary habits may also impact results across studies.

Regarding dietary behaviors, the frequency of fast-food consumption was unfavorably associated with adherence to PA and SB recommendations. Participants who avoided eating in fast-food restaurants had nearly twice the odds of fulfilling the PA and SB guidelines. Significant associations were primarily observed among boys for PA and girls for SB. Previous research offers ambiguous results on this association. Consistent with our findings, a recent study in Greece reported increased odds of frequent fast-food consumption among adolescents with prolonged screen time after adjusting for several covariates [73]. Likewise, a longitudinal US study identified positive associations between total screen-based sedentary time and consumption of low-nutrient foods, including fast-foods [74]. However, regarding PA, studies from Europe and Iran indicated an inverse association between PA levels and fast-food consumption among youth [20,75], while others report no significant associations [63,68]. Overall, these findings suggest an inverse relationship between unhealthy dietary patterns and adherence to global movement recommendations.

Moreover, our findings indicate a potential link between breakfast consumption frequency and adherence to sedentary lifestyle recommendations. A recent randomized controlled trial has shown reduced afternoon sedentary time among daily breakfast consumers compared to intermittent breakfast consumers [76]. Although data from the HELENA European study suggested a potential association between self-reported breakfast consumption and sedentary time in adolescents, the evidence was not strong since the results were not consistent across measurement methods or gender [77].

Finally, a strong association was also observed between sedentary time and food consumption in front of screens. Adolescents who rarely ate meals or snacks in front of screens had 6-fold higher odds of complying with the recommendations for total daily screen time. Previous studies have shown that reducing screen-time eating correlates with decreased energy intake and changes in food choices, particularly with increased consumption of unhealthy foods and decreased intake of fruits and vegetables [78,79,80]. TV viewing is the screen-based sedentary behavior most linked to nibbling, as the hands are generally free in contrast to other sedentary behaviors like console playing or computer use.

It is important to note that our analysis found no evidence of a relationship between adherence to PA and concurrent adherence to SB guidelines among adolescents, highlighting the distinct nature of these two behaviors. This observation is supported by recent reviews [81], while cluster analysis studies have also revealed mixed patterns of high PA and SB [24,26,82]; suggesting that adolescents may be active at certain times but sedentary during the remaining hours.

This study has several strengths. Among them are the large sample size and the use of a standardized, international protocol from an established multinational survey. Moreover, it is one of the relatively few studies that have investigated the complex relationship between diet, dietary behaviors, and PA and SB recommendations while controlling for important parameters such as the BMI, family affluence, and sleeping habits. Nevertheless, several limitations must also be acknowledged. The study relied on self-reported data, especially regarding the assessment of diet, PA, and SB, which, among others, may also lead to the introduction of social desirability bias [83,84]. The assessment of food consumption was based on a limited number of food items, whereas the diet quality score used in the present analysis was based on a few items and has not been formally validated. Additionally, the cross-sectional design precludes the establishment of a temporal relationship, while residual confounding cannot be excluded in observational studies despite controlling for various possible confounders. Last but not least, reported levels of PA and SB, as well as percentages of adherence to physical activity and sedentary behavior recommendations, pertain specifically to the 2017/2018 time period and should be interpreted accordingly.

## 5. Conclusions

In conclusion, in this large sample of adolescents, aspects of healthier dietary habits and healthier diet-related behaviors were associated with higher adherence to physical activity recommendations, higher adherence to recommendations for limiting screen time use, or both, thus highlighting their strong interconnection. Effective interventions and educational programs tailored specifically for children and adolescents, incorporating healthy dietary habits and movement behaviors, should be implemented, actively involving parents, educators, and health care providers. Additionally, since digital media currently forms an integral part of each child’s everyday life, it is essential to understand better its risks and benefits on the physical, emotional, and mental health of the children and promote healthy screen time habits [7,85,86]. Addressing all three behaviors in an integrated, holistic approach will contribute to the promotion of the health and well-being of adolescents and future adults.

## Figures and Tables

**Table 1:** Sociodemographic and baseline characteristics of the final sample consisted of 3357 students, who were participants in the Greek arm of the 2017/2018 Health Behavior of School-Aged Children Study (HBSC).

Characteristics	Total Sample, *n* (%)
Gender	
Male	1597 (47.6)
Female	1760 (52.4)
Age group	
11 years old	1019 (30.3)
13 years old	1164 (34.7)
15 years old	1174 (35.0)
Region/municipality	
Attica/Thessaloniki	1775 (52.9)
Other regions	1582 (47.1)
Place of birth	
Greece	3246 (96.7)
Other country	111 (3.3)
Parents’ place of birth	
Both parents born in Greece	2529 (75.3)
At least one parent born outside Greece	828 (24.7)
BMI groups (based on z-score) ^a^	
Non-overweight	2414 (71.9)
Overweight	780 (23.2)
Missing BMI	163 (4.9)
Family affluence scale groups (based on FAS score) ^b^	
Low 20% affluent	467 (13.9)
Middle 60% affluent	2164 (64.5)
High 20% affluent	726 (21.6)
MVPA ≥ 60 min (days/week), mean (sd)	4.0 (2.0)
Fulfillment of physical activity recommendations ^c^	
No	2817 (83.9)
Yes	540 (16.1)
Average daily sedentary time (minutes), mean (sd)	390.3 (237.4)
Fulfillment of sedentary time limit recommendation ^d^	
No	3027 (90.2)
Yes	330 (9.8)
Life satisfaction level, mean (sd) ^e^	7.5 (1.9)
Trying to lose weight at present	
No	2663 (79.3)
Yes	694 (20.7)
Adherence to sleep duration recommendations by age ^f^	
No	1804 (53.7)
Yes	1553 (46.3)

Abbreviations: standard deviation (sd); Body Mass Index (BMI); Μoderate to Vigorous Physical Activity (MVPA); ^a^ Overweight category stands for overweight and obese; non-overweight stands for underweight and normal weight students, according to 2007 WHO growth charts; ^b^ Tertiles were calculated based on FAS score distribution by gender and age group. ^c^ WHO guidelines for at least an average of 60 min per day of MVPA across the week. ^d^ Guidelines for a maximum of 120 min of sedentary time daily. ^e^ Values range from 0 to 10; Ten corresponds to the best possible life as reported by the student, and zero corresponds to the worst possible life. ^f^ Based on age-specific guidelines of the American Academy of Sleep Medicine.

**Table 2:** Sociodemographic and baseline characteristics of the final sample consisted of 3357 students, who were participants in the Greek arm of the 2017/2018 Health Behavior of School-Aged Children Study (HBSC), classified by fulfillment (no/yes) of the daily physical activity and sedentary time recommendations ^a,b^.

Characteristics	Fulfillment of Physical Activity Recommendations ^a^				Fulfillment of Sedentary Time Recommendations ^b^			
	No (*n* = 2817)	Yes (*n* = 540)	*p*-Value ^†^	Phi/ Cramér’s V ^††^	No (*n* = 3027)	Yes (*n* = 330)	*p*-Value ^†^	Phi/ Cramér’s V ^††^
Gender			<0.001 ***	–0.104			<0.001 ***	+0.060
Male	1276 (79.9)	321 (20.1)			1470 (92.1)	127 (7.9)		
Female	1541 (87.6)	219 (12.4)			1557 (88.5)	203 (11.5)		
Age group			<0.001 ***	0.092			<0.001 ***	0.198
11 years old	806 (79.1)	213 (20.9)			828 (81.3)	191 (18.7)		
13 years old	985 (84.6)	179 (15.4)			1093 (93.9)	71 (6.1)		
15 years old	1026 (87.4)	148 (12.6)			1106 (94.2)	68 (5.8)		
Region/municipality			0.278	+0.019			0.147	+0.025
Attica/Thessaloniki	1501 (84.6)	274 (15.4)			1613 (90.9)	162 (9.1)		
Other regions	1316 (83.2)	266 (16.8)			1414 (89.4)	168 (10.6)		
Place of birth			0.008 **	+0.046			0.345	–0.016
Greece	2734 (84.2)	512 (15.8)			2924 (90.1)	322 (9.9)		
Other country	83 (74.8)	28 (25.2)			103 (92.8)	8 (7.2)		
Parents’ place of birth			0.930	+0.002			0.004 **	–0.050
Both parents born in Greece	2123 (83.9)	406 (16.1)			2259 (89.3)	270 (10.7)		
At least one parent born outside Greece	694 (83.8)	134 (16.2)			768 (92.8)	60 (7.2)		
BMI groups (based on z-score) ^c^			0.013 *	0.051			0.375	0.024
Non-Overweight	2002 (82.9)	412 (17.1)			2166 (89.7)	248 (10.3)		
Overweight	681 (87.3)	99 (12.7)			713 (91.4)	67 (8.6)		
Missing BMI	134 (82.2)	29 (17.8)			148 (90.8)	15 (9.2)		
Family affluence scale groups (based on FAS score) ^d^			<0.001 ***	0.097			0.735	0.014
Low 20% affluent	403 (86.3)	64 (13.7)			417 (89.3)	50 (10.7)		
Middle 60% affluent	1854 (85.7)	310 (14.3)			1957 (90.4)	207 (9.6)		
High 20% affluent	560 (77.1)	166 (22.9)			653 (89.9)	73 (10.1)		
Life satisfaction level, mean (sd) ^e^	7.4 (1.9)	8.0 (1.8)	<0.001 ***		7.5 (1.9)	8.2 (1.9)	<0.001 ***	
Trying to lose weight at present			0.534	+0.011			0.373	–0.015
No	2240 (84.1)	423 (15.9)			2395 (89.9)	268 (10.1)		
Yes	577 (83.1)	117 (16.9)			632 (91.1)	62 (8.9)		
Adherence to sleep duration recommendations by age ^f^			0.625	+0.008			<0.001 ***	+0.115
No	1519 (84.2)	285 (15.8)			1684 (93.3)	120 (6.7)		
Yes	1298 (83.6)	255 (16.4)			1343 (86.5)	210 (13.5)		

Abbreviations: standard deviation (sd); Body Mass Index (BMI). ^a^ Guidelines for at least an average of 60 min per day of moderate-to-vigorous physical activity across the week. ^b^ Guidelines for a maximum of 120 min of sedentary time daily. ^c^ Overweight category stands for overweight and obese; non-overweight stands for underweight and normal weight students, according to 2007 WHO growth charts; ^d^ Tertiles were calculated based on FAS score distribution by gender and age group. ^e^ Values range from 0 to 10; Ten corresponds to the best possible life as reported by the students and zero corresponds to the worst possible life; ^f^ Based on age-specific guidelines of the American Academy of Sleep Medicine; ^†^ *p*-values from Pearson’s X^2^-test for categorical variables and two-sample Wilcoxon rank-sum test for the continuous variable; ^††^ The strength and direction of the association for 2 × 2 cross-tabulations were measured using Phi (φ) coefficient, whereas the strength of the association for contingency tables larger than 2 × 2 was measured with Cramér’s V coefficient; “*” *p*-value: <0.05, “**” *p*-value: <0.01 and “***” *p*-value: <0.001.

**Table 3:** Consumption of food groups, diet-quality, and diet-related behaviors of the final sample consisted of 3357 students, who were participants in the 2017/2018 Greek arm of the Health Behavior of School-Aged Children Study (HBSC), for the total sample and by fulfillment of the daily physical activity and sedentary time recommendations ^a,b^.

Food Groups/ Diet-Related Behaviors	Total Sample *n* (%)	Fulfillment of Physical Activity Recommendations ^a^				Fulfillment of Sedentary Time Recommendations ^b^			
		No (*n* = 2817)	Yes (*n* = 540)	*p*-Value ^†^	Phi/ Cramér’s V ^††^	No (*n* = 3027)	Yes (*n* = 330)	*p*-Value ^†^	Phi/ Cramér’s V ^††^
Fruit intake, days/week				<0.001 ***	0.173			<0.001 ***	0.112
≤1	645 (19.2)	581 (20.6)	64 (11.8)			603 (19.9)	42 (12.7)		
2–4	1133 (33.7)	1005 (35.7)	128 (23.7)			1050 (34.7)	83 (25.2)		
5–6	549 (16.4)	460 (16.3)	89 (16.5)			494 (16.3)	55 (16.7)		
≥7	1030 (30.7)	771 (27.4)	259 (48.0)			880 (29.1)	150 (45.4)		
Vegetable intake, days/week				<0.001 ***	0.106			0.005 **	0.062
≤1	626 (18.6)	537 (19.1)	89 (16.5)			576 (19.0)	50 (15.2)		
2–4	871 (26.0)	770 (27.3)	101 (18.7)			803 (26.5)	68 (20.6)		
5–6	719 (21.4)	611 (21.7)	108 (20.0)			644 (21.3)	75 (22.7)		
≥7	1141 (34.0)	899 (31.9)	242 (44.8)			1004 (33.2)	137 (41.5)		
Sweets intake, days/week				0.003 **	0.064			<0.001 ***	0.151
≥7	506 (15.1)	429 (15.2)	77 (14.3)			488 (16.1)	18 (5.4)		
5–6	438 (13.0)	373 (13.2)	65 (12.0)			412 (13.6)	26 (7.9)		
2–4	1128 (33.6)	974 (34.6)	154 (28.5)			1038 (34.3)	90 (27.3)		
≤1	1285 (38.3)	1041 (37.0)	244 (45.2)			1089 (36.0)	196 (59.4)		
SSBs intake, days/week				0.460	0.028			<0.001 ***	0.136
≥7	178 (5.3)	144 (5.1)	34 (6.3)			171 (5.6)	7 (2.1)		
5–6	160 (4.8)	138 (4.9)	22 (4.1)			152 (5.0)	8 (2.4)		
2–4	642 (19.1)	532 (18.9)	110 (20.4)			622 (20.6)	20 (6.1)		
≤1	2377 (70.8)	2003 (71.1)	374 (69.2)			2082 (68.8)	295 (89.4)		
Diet quality group ^c^				<0.001 ***	0.157			<0.001 ***	0.155
Good	612 (18.2)	443 (15.7)	169 (31.3)			498 (16.5)	114 (34.5)		
Moderate	1331 (39.7)	1123 (39.9)	208 (38.5)			1196 (39.5)	135 (41.0)		
Poor	1414 (42.1)	1251 (44.4)	163 (30.2)			1333 (44.0)	81 (24.5)		
Eating breakfast on weekdays				0.007 **	0.055			<0.001 ***	0.109
Never	940 (28.0)	808 (28.7)	132 (24.4)			881 (29.1)	59 (17.9)		
1–4 days a week	811 (24.2)	695 (24.7)	116 (21.5)			752 (24.8)	59 (17.9)		
5 days a week	1606 (47.8)	1314 (46.6)	292 (54.1)			1394 (46.1)	212 (64.2)		
Eating with family				0.501	+0.012			0.003 **	+0.051
Once a week or less	676 (20.1)	573 (20.3)	103 (19.1)			630 (20.8)	46 (13.9)		
Most days	2681 (79.9)	2244 (79.7)	437 (80.9)			2397 (79.2)	284 (86.1)		
Eating in fast-food restaurants				0.001 **	0.072			<0.001 ***	0.156
At least once a week	800 (23.8)	687 (24.4)	113 (20.9)			763 (25.2)	37 (11.2)		
1–3 days a month	1447 (43.1)	1214 (43.1)	233 (43.2)			1332 (44.0)	115 (34.9)		
Less than once a month	944 (28.1)	795 (28.2)	149 (27.6)			799 (26.4)	145 (43.9)		
Never	166 (5.0)	121 (4.3)	45 (8.3)			133 (4.4)	33 (10.0)		
Eating in front of screens ^d^				0.869	0.009			<0.001 ***	0.221
Most days	1108 (33.0)	934 (33.1)	174 (32.2)			1071 (35.4)	37 (11.2)		
Less often	1668 (49.7)	1399 (49.7)	269 (49.8)			1508 (49.8)	160 (48.5)		
Rarely	581 (17.3)	484 (17.2)	97 (18.0)			448 (14.8)	133 (40.3)		

Abbreviations: Sugar-sweetened beverages (SSBs). ^a^ Recommendations for at least an average of 60 min per day of moderate-to-vigorous physical activity across the week. ^b^ Recommendations for a maximum of 120 min of sedentary time daily; ^c^ Groups calculated using dietary score’s tertiles; poor (dietary scores 0–9), moderate (dietary scores 10–12), good (dietary scores 13–16); ^d^ Calculated using the combined score of eating snacks/meals in front of screens; Most days (score ≥ 4), less often (2 < score < 4), rarely (score ≤ 2) ^†^ *p*-values from Pearson’s X^2^-test for categorical variables; ^††^ The strength and direction of the association for 2 × 2 cross-tabulations was measured using Phi (φ) coefficient, whereas the strength of the association for contingency tables larger than 2 × 2 was measured with Cramér’s V coefficient; “**” *p*-value: <0.01 and “***” *p*-value: <0.001.

**Table 4:** Adjusted odds ratios (aOR) and associated 95% confidence intervals (CI) derived from multiple logistic regression models, which explore the associations of diet-related habits and behaviors, as well as baseline characteristics, with adherence to daily physical activity recommendations ^a^, among 3357 students, who were participants in the 2017/2018 Greek arm of the HBSC study. This is presented in the total sample and by gender (Model 1).

Characteristics/ Dietary Habits and Behaviors	Total Sample		Boys (*n* = 1597)		Girls (*n* = 1760)	
	Adjusted Model ^†^ aOR (95%CI)	*p*-Value	Adjusted Model ^†^ aOR (95%CI)	*p*-Value	Adjusted Model ^†^ aOR (95%CI)	*p*-Value
Gender						
Male	Ref					
Female	0.49 (0.40–0.62)	<0.001 ***				
Age group						
11 years old	Ref		Ref		Ref	
13 years old	0.80 (0.62–1.02)	0.076	0.95 (0.68–1.34)	0.779	0.65 (0.44–0.96)	0.031 *
15 years old	0.68 (0.53–0.87)	0.002 **	0.80 (0.57–1.12)	0.190	0.52 (0.35–0.79)	0.002 **
BMI z-score ^b^						
Non-overweight	Ref		Ref		Ref	
Overweight	0.59 (0.45–0.78)	<0.001 ***	0.62 (0.44–0.89)	0.009 **	0.52 (0.32–0.84)	0.008 **
Missing BMI	0.89 (0.56–1.41)	0.620	0.87 (0.46–1.61)	0.651	0.95 (0.47–1.93)	0.893
Family affluence scale (FAS) score ^c^						
Low 20% affluent	Ref		Ref		Ref	
Middle 60% affluent	0.97 (0.70–1.36)	0.883	1.29 (0.81–2.06)	0.271	0.67 (0.42–1.05)	0.083
High 20% affluent	1.49 (1.03–2.16)	0.035 *	2.01 (1.20–3.57)	0.009 **	1.02 (0.64–1.64)	0.929
Life satisfaction level ^d^	1.09 (1.02–1.17)	0.010 *	1.05 (0.96–1.14)	0.315	1.15 (1.03–1.28)	0.009 **
Trying to lose weight at present						
No	Ref		Ref		Ref	
Yes	1.36 (1.07–1.72)	0.013 *	1.22 (0.84–1.76)	0.285	1.59 (1.14–2.21)	0.007 **
Adherence to sleep duration recommendations by age ^e^						
No	Ref		Ref		Ref	
Yes	0.94 (0.76–1.16)	0.554	0.98 (0.75–1.27)	0.877	0.87 (0.63–1.19)	0.376
Fulfillment of sedentary time recommendations ^f^						
No	Ref		Ref		Ref	
Yes	1.19 (0.84–1.69)	0.331	1.42 (0.90–2.23)	0.126	1.03 (0.62–1.71)	0.904
Fruit intake, days/week						
≤1	Ref		Ref		Ref	
2–4	1.02 (0.73–1.43)	0.888	1.21 (0.76–1.92)	0.420	0.87 (0.53–1.43)	0.584
5–6	1.42 (0.95–2.09)	0.082	1.99 (1.19–3.31)	0.008 **	0.80 (0.42–1.51)	0.494
≥7	2.26 (1.62–3.17)	<0.001 ***	2.95 (1.92–4.53)	<0.001 ***	1.68 (1.02–2.73)	0.039 *
Vegetables intake, days/week						
≤1	Ref		Ref		Ref	
2–4	0.81 (0.58–1.14)	0.223	0.83 (0.54–1.28)	0.406	0.76 (0.42–1.39)	0.374
5–6	1.03 (0.75–1.43)	0.839	0.97 (0.64–1.46)	0.870	1.13 (0.63–2.04)	0.680
≥7	1.35 (1.00–1.82)	0.049 *	1.25 (0.82–1.90)	0.298	1.56 (0.96–2.55)	0.073
Sweets intake, days/week						
≥7	Ref		Ref		Ref	
5–6	0.98 (0.66–1.46)	0.926	0.74 (0.44–1.25)	0.260	1.43 (0.80–2.55)	0.220
2–4	0.82 (0.60–1.13)	0.219	0.80 (0.52–1.23)	0.309	0.85 (0.53–1.38)	0.517
≤1	1.12 (0.83–1.53)	0.454	1.10 (0.71–1.71)	0.661	1.15 (0.71–1.86)	0.571
SSBs, days/week						
≥7	Ref		Ref		Ref	
5–6	0.67 (0.37–1.21)	0.180	0.74 (0.32–1.70)	0.477	0.60 (0.24–1.52)	0.284
2–4	0.86 (0.52–1.44)	0.579	1.01 (0.53–1.93)	0.962	0.64 (0.28–1.48)	0.298
≤1	0.70 (0.44–1.12)	0.134	0.74 (0.40–1.37)	0.343	0.62 (0.39–1.28)	0.194
Eating breakfast on weekdays						
Never	Ref		Ref		Ref	
1–4 days a week	0.99 (0.75–1.29)	0.930	1.11 (0.73–1.68)	0.618	0.87 (0.59–1.28)	0.478
5 days a week	1.14 (0.91–1.43)	0.239	1.55 (1.12–2.14)	0.008 **	0.78 (0.55–1.11)	0.163
Eating with family						
Once a week or less	Ref		Ref		Ref	
Most days	0.83 (0.66–1.05)	0.123	0.91 (0.65–1.26)	0.560	0.72 (0.50–1.03)	0.079
Eating in fast-food restaurants						
At least once a week	Ref		Ref		Ref	
1–3 days a month	1.16 (0.89–1.52)	0.325	1.22 (0.87–1.71)	0.247	1.15 (0.73–1.80)	0.547
Less than once a month	0.99 (0.75–1.30)	0.789	0.94 (0.65–1.37)	0.759	1.08 (0.67–1.75)	0.734
Never	1.77 (1.14–2.75)	0.011 *	2.12 (1.15–3.88)	0.016 *	1.64 (0.82–3.29)	0.160
Eating in front of screens ^g^						
Most days	Ref		Ref		Ref	
Less often	1.00 (0.79–1.27)	0.986	1.10 (0.80–1.51)	0.537	0.88 (0.60–1.30)	0.521
Rarely	0.91 (0.65–1.28)	0.598	0.83 (0.53–1.33)	0.451	0.97 (0.59–1.60)	0.903

Abbreviations: Health Behavior in School-Aged Children (HBSC); Adjusted Odds Ratio (aOR); Confidence Interval (CI); Body Mass Index (BMI); Sugar-sweetened beverages (SSBs). ^a^ Recommendations for at least an average of 60 min per day of moderate-to-vigorous physical activity across the week. ^b^ Overweight category stands for overweight and obese; non-overweight stands for underweight and normal weight students, according to 2007 WHO growth charts. ^c^ Tertiles were calculated based on FAS score distribution by gender and age group. ^d^ Values range from 0 to 10; Ten corresponds to the best possible life as reported by the student and zero to the worst possible life. ^e^ Based on age-specific guidelines of the American Academy of Sleep Medicine; ^f^ Recommendations for maximum 120 min of sedentary time daily; ^g^ Calculated using the combined score of eating snacks/meals in front of screens; Most days (score ≥ 4), less often (2 < score < 4), rarely (score ≤ 2) ^†^ Adjusted model: for all variables included in the table as well as region, place of birth and parental place of birth. Analysis accounted for survey design. “*” *p*-value: <0.05, “**” *p*-value: <0.01 and “***” *p*-value: <0.001.

**Table 5:** Adjusted odds ratios (aOR) and associated 95% confidence intervals (CI) derived from multiple logistic regression models, which explore the associations of diet-related habits and behaviors, as well as baseline characteristics, with adherence to daily sedentary time recommendations ^a^, among 3357 students, who were participants in the 2017/2018 Greek arm of the HBSC study. This is presented in the total sample and by gender (Model 2).

Characteristics/ Dietary Habits and Behaviors	Total Sample		Boys (*n* = 1597)		Girls (*n* = 1760)	
	Adjusted Model ^†^ aOR (95%CI)	*p*-Value	Adjusted Model ^†^ aOR (95%CI)	*p*-Value	Adjusted Model ^†^ aOR (95%CI)	*p*-Value
Gender						
Male	Ref					
Female	1.43 (1.09–1.89)	0.011 *				
Age group						
11 years old	Ref		Ref		Ref	
13 years old	0.31 (0.22–0.44)	<0.001 ***	0.33 (0.20–0.53)	<0.001 ***	0.29 (0.18–0.47)	<0.001 ***
15 years old	0.35 (0.25–0.50)	<0.001 ***	0.62 (0.39–1.00)	0.051	0.20 (0.12–0.34)	<0.001 ***
BMI z-score ^b^						
Non-overweight	Ref		Ref		Ref	
Overweight	0.92 (0.65–1.29)	0.626	1.00 (0.64–1.59)	0.979	0.75 (0.44–1.28)	0.294
Missing BMI	0.77 (0.43–1.38)	0.384	0.52 (0.16–1.67)	0.266	0.94 (0.45–1.97)	0.878
Family affluence scale (FAS) score ^c^						
Low 20% affluent	Ref		Ref		Ref	
Middle 60% affluent	0.82 (0.59–1.15)	0.252	1.19 (0.66–2.15)	0.555	0.61 (0.37–0.98)	0.043 *
High 20% affluent	0.78 (0.50–1.20)	0.255	0.91 (0.45–1.82)	0.787	0.67 (0.37–1.22)	0.192
Life satisfaction level ^d^	1.08 (0.98–1.19)	0.099	1.22 (1.05–1.43)	0.011 *	0.99 (0.87–1.13)	0.924
Trying to lose weight at present						
No	Ref		Ref		Ref	
Yes	0.98 (0.69–1.40)	0.916	1.49 (0.86–2.60)	0.157	0.77 (0.47–1.25)	0.285
Adherence to sleep duration recommendations by age ^e^						
No	Ref		Ref		Ref	
Yes	2.03 (1.53–2.70)	<0.001 ***	1.74 (1.14–2.64)	0.010 *	2.40 (1.65–3.48)	<0.001 ***
Fulfilling physical activity recommendations ^f^						
No	Ref		Ref		Ref	
Yes	1.24 (0.87–1.77)	0.227	1.43 (0.90–2.26)	0.124	1.09 (0.64–1.84)	0.756
Fruit intake, days/week						
≤1	Ref		Ref		Ref	
2–4	0.98 (0.63–1.52)	0.932	1.17 (0.57–2.43)	0.662	0.80 (0.47–1.39)	0.434
5–6	1.17 (0.74–1.85)	0.505	1.14 (0.51–2.53)	0.743	1.12 (0.61–2.07)	0.701
≥7	1.35 (0.89–2.05)	0.153	1.19 (0.59–2.40)	0.633	1.40 (0.83–2.39)	0.208
Vegetables intake, days/week						
≤1	Ref		Ref		Ref	
2–4	0.86 (0.56–1.32)	0.483	0.78 (0.40–1.54)	0.475	0.89 (0.50–1.59)	0.701
5–6	1.05 (0.68–1.63)	0.808	1.08 (0.58–1.99)	0.807	1.12 (0.60–2.09)	0.709
≥7	0.90 (0.59–1.37)	0.613	0.99 (0.52–1.90)	0.978	0.88 (0.51–1.51)	0.646
Sweets intake, days/week						
≥7	Ref		Ref		Ref	
5–6	1.67 (0.87–3.18)	0.119	1.36 (0.49–3.77)	0.552	1.75 (0.77–4.00)	0.181
2–4	1.66 (0.95–2.90)	0.074	1.54 (0.64–3.71)	0.331	1.61 (0.79–3.31)	0.191
≤1	2.41 (1.43–4.04)	0.001 **	1.95 (0.85–4.48)	0.116	2.46 (1.25–4.85)	0.009 **
SSBs, days/week						
≥7	Ref		Ref		Ref	
5–6	0.92 (0.29–2.87)	0.885	0.56 (0.12–2.68)	0.457	1.56 (0.31–7.79)	0.584
2–4	0.47 (0.18–1.22)	0.119	0.38 (0.11–1.37)	0.128	0.60 (0.14–2.51)	0.482
≤1	0.96 (0.40–2.28)	0.928	1.00 (0.29–3.47)	0.952	1.06 (0.31–3.64)	0.925
Eating breakfast on weekdays						
Never	Ref		Ref		Ref	
1–4 days a week	1.16 (0.79–1.70)	0.454	1.34 (0.72–2.50)	0.354	1.02 (0.63–1.63)	0.944
5 days a week	1.83 (1.34–2.51)	<0.001 ***	1.70 (1.02–2.85)	0.043 *	1.86 (1.20–2.87)	0.005 **
Eating with family						
Once a week or less	Ref		Ref		Ref	
Most days	1.08 (0.74–1.56)	0.687	0.88 (0.50–1.55)	0.657	1.17 (0.70–1.94)	0.546
Eating in fast-food restaurants						
At least once a week	Ref	Ref	Ref		Ref	
1–3 days a month	1.06 (0.69–1.64)	0.773	0.72 (0.38–1.34)	0.350	1.63 (0.88–3.02)	0.119
Less than once a month	1.48 (0.98–2.22)	0.059	1.11 (0.67–1.84)	0.614	2.04 (1.10–3.77)	0.023
Never	1.95 (1.11–3.43)	0.020 *	1.37 (0.59–3.15)	0.323	2.74 (1.22–6.15)	0.015 *
Eating in front of screens ^g^						
Most days	Ref	Ref	Ref		Ref	
Less often	2.31 (1.52–3.52)	<0.001 ***	3.32 (1.80–6.12)	<0.001 ***	1.67 (0.91–3.07)	0.096
Rarely	5.79 (3.67–9.14)	<0.0001 ***	6.01 (3.08–11.72)	<0.001 ***	5.92 (3.16–11.11)	<0.001 ***

Abbreviations: Health Behavior in School-Aged Children (HBSC); Adjusted Odds Ratio (aOR); Confidence Interval (CI); Body Mass Index (BMI); Sugar-sweetened beverages (SSBs). ^a^ Recommendations for a maximum of 120 min of sedentary time daily. ^b^ Overweight category stands for overweight and obese; non-overweight stands for underweight and normal weight students, according to 2007 WHO growth charts. ^c^ Tertiles were calculated based on FAS score distribution by gender and age group. ^d^ Values range from 0 to 10; Ten corresponds to the best possible life as reported by the student and zero to the worst possible life. ^e^ Based on age-specific guidelines of the American Academy of Sleep Medicine; ^f^ Recommendations for at least an average of 60 min per day of moderate-to-vigorous physical activity across the week. ^g^ Calculated using the combined score of eating snacks/meals in front of screens; Most days (score ≥ 4), less often (2 < score < 4), rarely (score ≤ 2). ^†^ Adjusted model: for all variables included in the table as well as region, place of birth, and parental place of birth. Analysis accounted for survey design. “*” *p*-value: <0.05, “**” *p*-value: <0.01 and “***” *p*-value: <0.001.

**Table 6:** Adjusted odds ratios (aOR) and associated 95% confidence intervals (CI) derived from multiple logistic regression models, which explore the association of diet quality groups with adherence to daily physical activity recommendations ^a^ and daily sedentary time recommendations ^b^ among 3357 students, who were participants in the Greek arm of the HBSC study, in the total sample and by gender (Model 3).

Diet Quality Group	Total Sample		Boys (*n* = 1597)		Girls (*n* = 1760)	
	Adjusted Model ^†^ aOR (95%CI)	*p*-Value	Adjusted Model ^†^ aOR (95%CI)	*p*-Value	Adjusted Model ^†^ aOR (95%CI)	*p*-Value
Physical activity recommendations						
Diet quality group ^c^						
Good	Ref		Ref		Ref	
Moderate	0.49 (0.39–0.63)	<0.001 ***	0.50 (0.36–0.70)	<0.001 ***	0.44 (0.31–0.64)	<0.001 ***
Poor	0.37 (0.28–0.49)	<0.001 ***	0.35 (0.24–0.51)	<0.001 ***	0.38 (0.24–0.60)	<0.001 ***
Sedentary behavior recommendations						
Diet quality group ^c^						
Good	Ref		Ref		Ref	
Moderate	0.79 (0.60–1.06)	0.114	0.91 (0.53–1.55)	0.724	0.73 (0.49–1.07)	0.107
Poor	0.67 (0.47–0.94)	0.023 *	0.71 (0.39–1.27)	0.245	0.63 (0.41–0.97)	0.036 *

Abbreviations: Health Behavior in School-Aged Children (HBSC); Adjusted Odds Ratio (aOR); Confidence Interval (CI); ^a^ Recommendations for at least an average of 60 min per day of moderate-to-vigorous physical activity across the week; ^b^ Recommendations for maximum 120 min of sedentary time daily; ^c^ Groups calculated using dietary score’s tertiles; poor (dietary scores 0–9), moderate (dietary scores 10–12), good (dietary scores 13–16). ^†^ Adjusted Model 3: includes the following independent variables as potential confounders: age groups, gender (only for the total sample), BMI z-scores groups (categorically; non-overweight, overweight, missing), FAS score groups (categorically; low, middle, high group), life satisfaction level (continuously, per 1 point in scale), trying to lose weight (no/yes), adherence to sleep duration recommendations by age (no/yes), fulfillment of sedentary behavior recommendations (when the dependent variable was physical activity recommendations), fulfillment of physical activity recommendations (when the dependent variable was sedentary behavior recommendations), region, student’s place of birth (Greece/other), parental place of birth (both parents born in Greece/at least one parent born outside Greece) and diet-related behaviors (eating breakfast on weekdays, eating with the family, eating in fast-food restaurants, eating in front of screens). Analysis accounted for survey design. “*” *p*-value: <0.05, “***” *p*-value: <0.001.

## Data Availability

The dataset analyzed in this study is available from the Epidemiology and Psychosocial Research Unit of the University Mental Health, Neurosciences, & Precision Medicine Research Institute “Costas Stefanis” (UMHRI) (erevnaHSBC@epipsi.gr) upon reasonable request.
